# Multi-centre national audit of juvenile localised scleroderma: describing current UK practice in disease assessment and management

**DOI:** 10.1186/s12969-018-0295-0

**Published:** 2018-12-18

**Authors:** Hanna Lythgoe, Beverley Almeida, Joshua Bennett, Chandrika Bhat, Amarpal Bilkhu, Mary Brennan, Samundeeswari Deepak, Pamela Dawson, Despina Eleftheriou, Kathryn Harrison, Daniel Hawley, Eleanor Heaf, Valentina Leone, Ema Long, Sarah Maltby, Flora McErlane, Nadia Rafiq, Athimalaipet V. Ramanan, Phil Riley, Satyapal Rangaraj, Giulia Varnier, Nick Wilkinson, Clare E. Pain

**Affiliations:** 1 Department of Paediatric Rheumatology, Alder Hey Children’s NHS Foundation Trust, Eaton Road, Liverpool, L12 2AP UK; 2 NIHR Alder Hey Clinical Research Facility, Alder Hey Children’s NHS Foundation Trust, Liverpool, UK; 3 Department of Women’s and Children’s, Institute of Translational Medicine, University of Liverpool, Liverpool, UK; 4 Department of Paediatric Rheumatology, Great North Children’s Hospital, Newcastle upon Tyne, UK; 5 Department of Paediatric Rheumatology, Bristol Royal Hospital for Children, Bristol, UK; 6 Department of Paediatric Rheumatology, Royal Hospital for Sick Children, Edinburgh, UK; 7 Department of Rheumatology, Nottingham Children’s Hospital, Nottingham University Hospital NHS Trust, Nottingham, UK; 8 Department of Rheumatology, Birmingham Children’s Hospital, Birmingham, UK; 9 Department of Paediatric Rheumatology, Great Ormond Street Hospital for Children NHS Foundation Trust, London, UK; 10 Department of Paediatric Rheumatology, Sheffield Children’s Hospital, Sheffield, UK; 11 Department of Paediatric Rheumatology, Royal Manchester Children’s Hospital, Manchester, UK; 12 Department of Paediatric Rheumatology, Leeds Children’s Hospital, Leeds Teaching Hospitals NHS Trust, Leeds, UK; 13 Department of Paediatric Rheumatology, Bristol Royal Hospital for Children & Bristol Medical School, University of Bristol, Bristol, UK; 14 Department of Rheumatology and Chronic Pain, Evelina London Children’s Hospital, Guy’s and St Thomas’s NHS Foundation Trust, London, UK

**Keywords:** Juvenile localised scleroderma, Morphoea, Paediatric, United Kingdom, Assessment, Management, Treatment, Audit

## Abstract

**Objective:**

To describe current United Kingdom practice in assessment and management of patients with juvenile localised scleroderma (JLS) compared to Paediatric Rheumatology European Society (PRES) scleroderma working party recommendations.

**Methods:**

Patients were included if they were diagnosed with JLS and were under the care of paediatric rheumatology between 04/2015–04/2016. Retrospective data was collected in eleven UK centres using a standardised proforma and collated centrally.

**Results:**

149 patients were included with a median age of 12.5 years. The outcome measures recommended by the PRES scleroderma working party were not utilised widely. The localised scleroderma cutaneous assessment tool was only used in 37.6% of patients. Screening for extracutaneous manifestations did not meet recommendations that patients with head involvement have regular screening for uveitis and baseline magnetic resonance imaging (MRI) brain: only 38.5% of these patients were ever screened for uveitis; 71.2% had a MRI brain.

Systemic treatment with disease-modifying anti-rheumatic drugs (DMARDs) or biologics was widely used (96.0%). In keeping with the recommendations, 95.5% of patients were treated with methotrexate as first-line therapy. 82.6% received systemic corticosteroids and 34.2% of patients required two or more DMARDs/biologics, highlighting the significant treatment burden. Second-line treatment was mycophenolate mofetil in 89.5%.

**Conclusion:**

There is wide variation in assessment and screening of patients with JLS but a consistent approach to systemic treatment within UK paediatric rheumatology. Improved awareness of PRES recommendations is required to ensure standardised care. As recommendations are based on low level evidence and consensus opinion, further studies are needed to better define outcome measures and treatment regimens for JLS.

## Introduction

The hallmark of JLS is chronic inflammation in the skin and soft tissues leading to fibrosis and eventually atrophy. This process can lead to complications such as joint contractures, limb length discrepancy and facial atrophy. The disease can have significant psychological and functional impact [1, 2]. However, the disease is not just confined to skin and soft tissue. Around 22% of patients may have extracutaneous manifestations including neurological, musculoskeletal and ocular complications [3].

Optimal treatment for JLS is not known and there have been few studies published [4–9]. First line treatment for active JLS is often methotrexate, its use supported by a randomised placebo controlled trial (RCT) and some observational studies [4–8]. Mycophenolate mofetil (MMF) is often used, although evidence is restricted to small case series [9]. A wide range of other treatments are used off–label in refractory cases with some suggestion of benefit [10–12]. Corticosteroids are used in the majority of patients presenting or relapsing with active JLS but there is wide variation of steroids regimens used [10, 11].

The Paediatric Rheumatology European Society (PRES) working party for scleroderma has produced recommendations for both diagnosis, assessment and management of JLS [13]. The aim of these recommendations was to provide consensus-based guidelines for a disease area with limited evidence base.

The aim of this national audit was to describe UK current practice in assessment and management of JLS in the context of PRES working party recommendations.

## Patients and methods

Patients were included if they had a diagnosis of JLS, were under 19 years at time of audit, and were under the care of a paediatric rheumatologist between April 2015 and April 2016. UK Paediatric Rheumatology tertiary centres (*n* = 14) were invited to take part and 11 centres participated (Birmingham, Bristol, Edinburgh, Leeds, Liverpool, London (Evelina London Children’s Hospital and Great Ormond Street Hospital), Manchester, Newcastle, Nottingham, Sheffield).

PRES working party recommendations for assessment and management of JLS [13] were used as audit standards. A standardised Microsoft Excel proforma was used to collect relevant retrospective data regarding demographics, disease, assessment, screening, management and outcomes.

Data were analysed descriptively with numerical data presented as medians and interquartile ranges (IQR), and categorical data as frequencies.

In accordance with the UK National Health Service Health Research Authority guidelines, ethical approval was not required as this was a clinical audit.

## Results

### Demographic data and diagnoses

In total 149 patients were identified and included with a female to male ratio of 1.9:1. Median age at diagnosis was 7.2 years (IQR 5.0–10.0 years) and at time of audit was 12.5 years (IQR 9.5–15 years).

The majority of patients (100/149) had linear scleroderma with 55/100 affecting the limb and 45/100 affecting the head/face. Of the remaining 49 patients, 22/49 had plaque morphoea (17/22 defined as superficial and 5/22 as deep); 15/49 had generalised morphoea; 2/49 had pansclerotic disease; 10/49 had mixed disease. Details of the anatomical area affected were not available.

### Extracutaneous manifestations

A total of 10/149 (6.7%) patients had ophthalmic involvement. Of these 3/10 had uveitis including one patient with non-facial JLS and two patients with associated eyelid/eyelash involvement; 1/10 had episcleritis (with associated eyelid/eyelash involvement) and 6/10 had eyelid/eyelash involvement with no uveitis or episcleritis.

There were 8/149 patients (5.4%) with neurological involvement: 2/8 had central nervous system (CNS) vasculitis (both had seizures and abnormal MRI and electroencephalograms, one patient also had headaches); 1/8 had seizures, headaches and abnormal MRI without identified CNS vasculitis; 5/8 patients suffered headaches in the absence of identified CNS vasculitis or seizures. Six of the eight patients with neurological involvement had linear scleroderma of the head/face with associated en coup de sabre.

There were 6/149 patients (4.0%) with synovitis: 4/6 had oligoarthritis at the site of skin lesion; 1/6 had polyarthritis which included the site of the lesion and 1/6 had oligoarthritis away from the site of the lesion.

The majority of patients were described as having deep involvement of subcutaneous tissue, muscle or bone: 113/149 (75.8%) had subcutaneous involvement, 27/149 (18.1%) had muscle involvement and 16/149 (10.7%) had bone involvement. Joint contractures were seen in 30/149 (20.1%) patients with 20/149 (13.4%) having limb length discrepancies; 15/149 (10.1%) had gait abnormality and 11/149 (7.4%) had an impact on hand function. There were 14/149 (9.4%) patients with involvement of the dental/oral area and of those five had temporomandibular joint involvement. Four patients (2.7%) had involvement of the breast area.

### Assessment of disease activity

Table 1 shows the PRES scleroderma working party recommendations our study population was audited against, together with the number of patients who met each of the recommendations.

**Table 1:** PRES scleroderma working group recommendations assessed within UK audit

Recommendation	Number of patients meeting recommendation (%)
Extra-cutaneous manifestations	
In craniofacial JLS, brain MRI is recommended	37/52 (71.2%)
In craniofacial JLS, uveitis screening every 6 months is recommended for the first 4 years of the disease	1/52 (1.9%)
In non-facial JLS, uveitis screening every 12 months is recommended for the first four years	6/97 (6.2%)
Dental assessment is suggested for any child with craniofacial JLS	12/52 (23.1%)
Assessment	
All patients with JLS, except for those with solitary small superficial plaque scleroderma, should be seen at baseline and minimum of every 12 months by a paediatric rheumatologist and (paediatric) dermatologist, ideally in a combined clinic	107/149 (71.8%)^a^
PGA-A, PGA-D and the LoSCAT, are acceptable tools for capturing cutaneous disease activity and damage	83/149 (55.7%)^b^
PROMs should be considered as an adjunct to clinical outcome measures and the following are recommended for serial measurements: Child/parent global assessment of disease severity on a visual analogue scale of 0–10 cm; Children’s Dermatology Life Quality Index	86/149 (57.7%)^c^
Treatment	
All types of active JLS require systemic immunosuppression, except for circumscribed small superficial non-progressive lesions, which are not crossing a joint and occur in non-cosmetically sensitive areas, where topical treatments alone may be appropriate	143/143 (100%)
First line treatment should be with methotrexate 15 mg/m^2^/week, max 25 mg/week either orally or subcutaneously	127/133 (95.5%)^e^
Bridging therapy with glucocorticoids should be used for patients requiring immunosuppression, particularly in rapidly progressive and severe disease, such as lesions crossing the joints and cosmetically disfiguring disease.	123/143 (86.0%)
Physiotherapy and/or occupational therapy are recommended for any patients with decreased range of motion in any joints	24/30 (80.0%)^d^

*JLS* Juvenile localised scleroderma, *PGA-A* Physician global assessment-activity, *PGA-D* Physician global assessment-damage, *LoSCAT* Localised scleroderma cutaneous activity tool, *PROM* Patient-reported outcome measures, *MMF* Mycophenalate mofetil, *ETN* Etanercept, *ANA* Anakinra, *TCZ* Tocilizumab

^a^Audit only recorded whether shared care clinic dermatology/rheumatology

^b^Patients where at least one of PGA-A, PGA-D or LoSCAT was used

^c^Patients where at least one form of PROM was used

^d^Patients with joint contractures

^e^ data unavailable for 10 patients

#### Use of assessment tools and imaging

Use of physician global assessment of activity (PGA-A) [14] occurred in 81/149 (54.4%) and localised scleroderma cutaneous assessment tool (LoSCAT) [14] in 56/149 (37.6%). Thermography was used in 34/149 (22.8%); ultrasound in 11/149 (7.4%); laser doppler in 5/149 (3.4%).

#### Screening for extra-cutaneous manifestations

Uveitis screening was performed in 35/149 (23.5%) of patients: 13/35 (37.1%) had a single screen; 18/35 (51.4%) had screening at regular intervals; 4/35 (11.4%) had screening on more than one occasion at irregular intervals.

Of those with known craniofacial involvement, only one patient received uveitis screening as frequently as recommended, 11/52 (21.2%) received annual screening and 32/52 (61.5%) were never screened.

### Management

Table 2 summarises treatments used in our population.

**Table 2:** Table summarising treatments used

		Systemic treatments								Systemic CS		Topical treatments		
		MTX	MMF	TCZ	ADA	AZA	ETN	ANK	CiC	IV	PO	CS	Vit D	Tac
Current treatment	No of patients	63	30	8	3	1	0	1	0	8	3	4	3	4
	Median duration treatment months (IQR)	16.0 (9.3–24)	13.0 (6.0–25.5)	17.5 (15.8–24.0)	26.3	14	n/a	31	n/a	7.0 (3.5–10)	9.3			
Previous treatment	No of patients	79	17	0	0	0	4	0	2	106	49	48	14	22
	Remission^a^	34	5	n/a	n/a	n/a	0	n/a	0	103^a^	45^a^			
	Inefficacy	8	6	n/a	n/a	n/a	3	n/a	2	0	3			
	Intolerance	37	6	n/a	n/a	n/a	1	n/a	0	3	1			
Never given	No of patients	7	102	141	146	148	145	148	147	35	97	97	132	123

*MTX* Methotrexate, *MMF* Mycophenolate mofetil, *TCZ* Tocilizumab, *ADA* Adalimumab, *AZA* Azathioprine, *ETN* Etanercept, *ANK* Anakinra, *CiC* Ciclosporin, *IV* Intravenous, *PO* Oral, *CS* Corticosteroid, *Vit D* Vitamin D analogue, *Tac* Tacrolimus, *IQR* Interquartile range

^a^Patients treated with steroids were often given a standard treatment course whilst being established on a DMARD/biologic which is stopped after a standard interval according to local protocol rather than remission

The indication for commencement of a biological was not captured in this audit. A biological may have been commenced for refractory skin involvement or extra-cutaneous manifestations such as uveitis, CNS involvement or synovitis

#### Systemic treatment

All but six patients were managed with systemic treatments (Table 2); of these six patients, three patients had superficial plaque morphoea managed with topical therapy alone and three patients had inactive disease.

Methotrexate was given as the first-line treatment in 127/133 (95.5%); information on the first-line DMARD was unknown in 10 patients. MMF was the most commonly used second-line treatment (34/38, 89.5%). A number of other DMARDs/biologics were used as second, and third line agents. No patients in our cohort received abatacept.

#### Use of allied health professionals

A total of 44/149 (29.5%) patients received physiotherapy and 26/149 (17.4%) received occupational therapy at least once. 29/149 (19.5%) had been seen by a psychologist at some point during their disease course.

#### Other treatments

Light therapy (either psoralen+UVA or UVA or UVB) was given to 5/149 (3.4%) patients of which four had linear disease (3 limb, 1 facial) and one had generalised morphoea. Light therapy was not used as recommended in all five patients (recommended for extending circumscribed superficial lesions; preferentially in children above the age of 12; preferably UVA-1 or UVB narrowband, PUVA therapy should be avoided).

17/149 (11.4%) had had surgery including fat transplants (10/149) and limb lengthening surgery (3/149). Data was insufficient to identify whether disease was inactive at this stage.

#### Outcomes

Over two-thirds of patients had achieved inactive disease at the point of review (108/149, 72.5%). Just over half (63/108, 58.3%) were still on systemic treatment. Additionally; 69/148 (46.6%) of patients had had at least one episode of drug free remission (data were missing for one patient).

## Discussion

This is the largest study to date to systematically audit practice for assessment and treatment of JLS in the UK. It highlights the significant disease and treatment burden for JLS patients within the UK.

Assessment of disease is primarily by clinician assessment. Use of other more standardised assessment tools, such as LoSCAT, is much more variable. The LoSCAT is a widely available bedside tool which has been validated in JLS [15]. Further training for paediatric rheumatologists and dermatologists on the LoSCAT may facilitate wider use of this tool and provide more standardised assessment of disease activity and damage.

Inactive disease was not defined for this study as a standardised approach to assessment was not seen across sites. Therefore, inactive disease was based on clinician opinion on note review. In 83/149 cases this included mLoSSI and/or PGA-A scoring.

Screening for CNS and eye involvement in those with craniofacial lesions is often not performed in the UK despite PRES working party recommendations. This may lead to under-recognition of extra-cutaneous features. However, MRI scans were performed more often in patients with known craniofacial involvement diagnosed after the implementation of the guideline (22/29) compared to those diagnosed prior (15/23). Where uveitis screening is performed there is no consistency in the frequency, and screening does not meet PRES working party recommendations. There may be a number of reasons for this omission, including possible limited resources, limited awareness of ophthalmology services and lack of evidence on the risk of uveitis in JLS [13]. Uveitis screening recommendations are largely consensus driven. Pending further studies to define uveitis rates, the authors agree that the PRES consensus based recommendations on uveitis screening should be followed within the UK.

This work highlights the significant treatment burden to JLS patients with 96.0% requiring treatment with at least one DMARD or biologic therapy and 34.2% receiving two or more. This finding may in part be due to selection bias, as this study only reviewed patients managed by paediatric rheumatologists while those with milder disease responding to topical treatment alone may be managed by a dermatologist alone.

Previous studies have highlighted the difference in practices between paediatric rheumatologists and dermatologists [14]. Shared care review by paediatric rheumatologists and dermatologists is widely practiced in the UK as recommended. Such collaborative care should greatly benefit patients by standardising management, and provide both rheumatology expertise, particularly related to extra-cutaneous manifestations, and dermatology expertise such as skin examination skills. Additionally, combined clinics may provide greater opportunity to consider topical treatments and phototherapy, which may be appropriate in some patients but with which many paediatric rheumatologists have little experience.

There is a widely consistent approach to use of systemic treatments for JLS within the UK. Most patients are managed with methotrexate as first-line treatment with bridging corticosteroid treatment as suggested by the PRES working group. However, intolerance leading to change in therapy remains a significant problem and is much higher in this cohort than reported in clinical studies in JLS [4, 16]. Whilst MMF is most often used as second-line treatment there remains limited evidence as to whether it is the most appropriate medication. Work is needed to determine its efficacy in JLS [9]. This study did not assess the details of steroid regimes in different centres; however, it did demonstrate variation between intravenous and oral corticosteroid use as demonstrated in previous studies [11].

Light-based therapies are only recommended for patients in a very small subgroup of patients with mild disease by the PRES scleroderma working group. This is supported by a recent meta-analysis comparing methotrexate and UVA/PUVA which demonstrated both had favourable effect but methotrexate was significantly superior to UVA/PUVA [17]. Four of the five children who received light therapy had received 2 or more DMARDs/biologics and had widespread and refractory disease. The use of light-based therapies in this group is not covered by these recommendations or by the aforementioned meta-analysis. Light based therapies, used in conjunction with a dermatologist with expertise, may have a role in patients with refractory and more extensive disease.

Physiotherapy, occupational therapy and psychological therapy are not widely used in JLS patients in the UK. Almost half of patients were not assessed using PROMs. Wider use of PROMs, including quality of life scores such as the Children’s Dermatology Life Quality Index (CDLQI) [18] may aid clinician assessment of the impact of the disease on patients. The use of PROMs could highlight those patients who would benefit from input of allied health professionals, leading to improved function and quality of life for these patients.

This study is limited by only reviewing JLS patients managed by paediatric rheumatologists. Further work to understand how many patients are managed elsewhere would help define the wider spectrum of disease. Whilst data was collected from a large number of centres and this is a significant strength of the study, it was not possible to collect data from all tertiary paediatric rheumatology centres across the UK.

Lack of awareness of the PRES working party recommendations is likely to have impacted on the findings of this study. Upcoming European recommendations on management of JLS via the Single Hub and Access point for paediatric Rheumatology in Europe (SHARE) initiative are in progress, which are likely to support a more standardized approach to disease assessment and management [19]. Such recommendations should lead to more equitable care and facilitate further studies to improve our understanding of this disease.

## Conclusion

In summary, this study demonstrates the significant disease and treatment burden for JLS patients in the UK. It shows a wide variation in the assessment and screening of JLS patients, but a consistent approach to systemic treatment within the UK with care of the majority of patients shared by paediatric rheumatologists and dermatologists.

Improved awareness of PRES recommendations and future SHARE recommendations is required to ensure standardised care. As such recommendations can now only be based on low level evidence and consensus opinion, further studies are needed to better define outcome measures and treatment regimens for JLS.

## Abbreviations

ADA: Adalimumab
ANK: Anakinra
AZA: Azathioprine
CDLQI: Children’s Dermatology Life Quality Index
CiC: Ciclosporin
CNS: Central nervous system
CS: Corticosteroid
DMARD: Disease-modifying anti-rheumatic drugs
ETN: Etanercept
IQR: Interquartile range
IV: Intravenous
JLS: Juvenile localised scleroderma
LoSCAT: Localised scleroderma cutaneous activity tool
MMF: Mycophenolate mofetil
MRI: Magnetic resonance imaging
MTX: Methotrexate
PGA-A: Physician global assessment – activity
PGA-D: Physician global assessment – damage
PO: Oral
PRES: Paediatric Rheumatology European Society
PROM: Patient-reported outcome measures
RCT: Randomised placebo controlled trial
SHARE: Single Hub and Access point for paediatric Rheumatology in Europe
Tac: Tacrolimus
TCZ: Tocilizumab
Vit D: Vitamin D analogue
